# Supplementary figures and images for: Relative contribution of COVID-19 vaccination and SARS-CoV-2 infection to population-level seroprevalence of SARS-CoV-2 spike antibodies in a large integrated health system
Source: PLoS One. 2024 Jun 20;19(6):e0303303. doi: 10.1371/journal.pone.0303303 (PMC11189213; doi:10.1371/journal.pone.0303303)

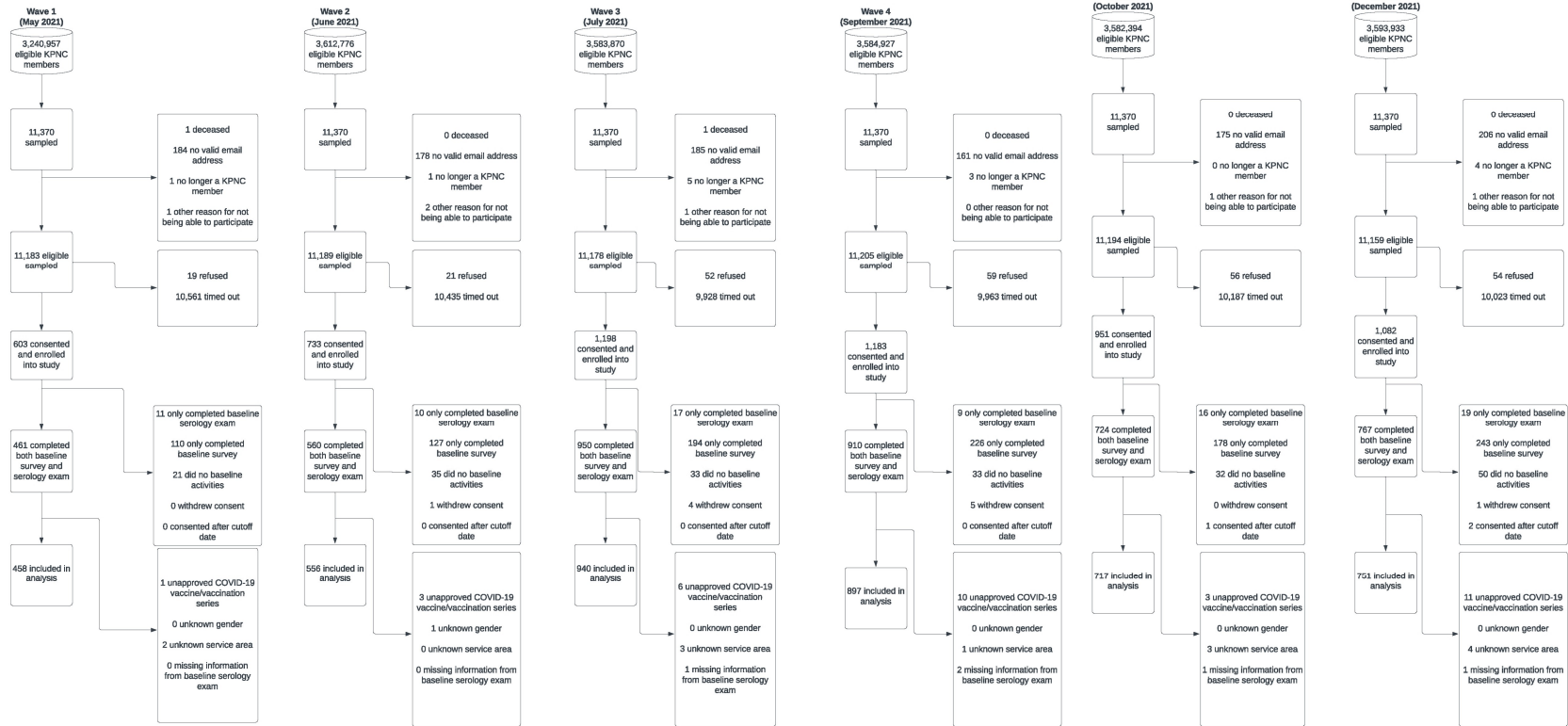

Supplement: S1 Fig — To be eligible for sampling, members needed to be enrolled in Kaiser Permanente Northern Calfornia membership for at least two months prior to the sampling date, be at least 7 years old on the sampling date, not listed in the “Do not contact” file for study participation, and had not been invited to a previous to a previous Kaiser Permanente Northern California COVID-19 serology study. Wave 1 only included English speaking members. (PDF) [file pone.0303303.s001.pdf]
