# Supplementary material for: Relative contribution of COVID-19 vaccination and SARS-CoV-2 infection to population-level seroprevalence of SARS-CoV-2 spike antibodies in a large integrated health system
Source: PLoS One. 2024 Jun 20;19(6):e0303303. doi: 10.1371/journal.pone.0303303 (PMC11189213; doi:10.1371/journal.pone.0303303)
Supplement: S1 Table — (DOCX) [file pone.0303303.s002.docx]

**Supplementary Table 1. Characteristics of the eligible Kaiser Permanente Northern California (KPNC) population May 2021 – April 2022 and non-respondent sampled population compared to respondent population before and after weighting.**

|  | **Before non-response weighting** | | | **After non-response weighting** | | | **Comparison with KPNC population** | | |
| --- | --- | --- | --- | --- | --- | --- | --- | --- | --- |
| **Characteristic** | **Non-respondent population**, N = 63,269 | **Respondent population**, N = 4,319 | **SMD** | **Non-respondent population**, N = 63,269 | **Respondent population**, N = 4,319 | **SMD** | **Eligible KPNC population**, N = 3,498,764 | **Respondent population**, N = 3,571,275 | **SMD** |
| **Age group (years)** |  |  | 0.64 |  |  | 0.14 |  |  | 0.10 |
| 7-17 | 6,319 (10%) | 72 (1.7%) |  | 6,319 (10%) | 72 (7.0%) |  | 333,650 (9.5%) | 250,210 (7.0%) |  |
| 18-44 | 27,250 (44%) | 1,062 (25%) |  | 27,250 (44%) | 1,062 (41%) |  | 1,478,525 (42%) | 1,467,581 (41%) |  |
| 45-64 | 18,943 (29%) | 1,743 (40%) |  | 18,943 (29%) | 1,743 (32%) |  | 1,051,877 (30%) | 1,155,306 (32%) |  |
| 65+ | 10,757 (17%) | 1,442 (34%) |  | 10,757 (17%) | 1,442 (20%) |  | 634,712 (18%) | 698,178 (20%) |  |
| **Sex** |  |  | 0.23 |  |  | 0.07 |  |  | 0.06 |
| Female | 33,239 (52%) | 2,751 (63%) |  | 33,239 (52%) | 2,751 (56%) |  | 1,840,164 (53%) | 1,983,715 (56%) |  |
| Male | 30,030 (48%) | 1,568 (37%) |  | 30,030 (48%) | 1,568 (44%) |  | 1,658,599 (47%) | 1,587,559 (44%) |  |
| **Race/Ethnicity** |  |  | 0.65 |  |  | 0.10 |  |  | 0.06 |
| Asian | 11,595 (21%) | 493 (12%) |  | 11,595 (21%) | 493 (21%) |  | 720,161 (21%) | 750,752 (21%) |  |
| Black | 11,899 (7.0%) | 352 (2.8%) |  | 11,899 (7.0%) | 352 (6.0%) |  | 234,344 (6.7%) | 215,024 (6.0%) |  |
| Hispanic | 11,777 (21%) | 371 (8.9%) |  | 11,777 (21%) | 371 (19%) |  | 690,766 (20%) | 662,280 (19%) |  |
| Other | 4,963 (10%) | 156 (4.4%) |  | 4,963 (10%) | 156 (8.9%) |  | 346,479 (9.9%) | 318,404 (8.9%) |  |
| White | 23,035 (41%) | 2,947 (71%) |  | 23,035 (41%) | 2,947 (45%) |  | 1,507,014 (43%) | 1,624,815 (45%) |  |
| **Geographic Area** |  |  | 0.21 |  |  | 0.06 |  |  | 0.05 |
| Central Valley | 7,361 (12%) | 326 (7.2%) |  | 7,361 (12%) | 326 (9.9%) |  | 401,410 (11%) | 355,054 (9.9%) |  |
| East Bay | 18,319 (27%) | 1,169 (27%) |  | 18,319 (27%) | 1,169 (28%) |  | 950,281 (27%) | 1,000,824 (28%) |  |
| North Bay | 3,941 (6.8%) | 458 (11%) |  | 3,941 (6.8%) | 458 (6.9%) |  | 248,204 (7.1%) | 244,676 (6.9%) |  |
| North Valley | 17,485 (27%) | 1,220 (28%) |  | 17,485 (27%) | 1,220 (27%) |  | 936,180 (27%) | 973,315 (27%) |  |
| Peninsula | 7,006 (12%) | 516 (12%) |  | 7,006 (12%) | 516 (12%) |  | 411,419 (12%) | 410,996 (12%) |  |
| South Bay | 9,157 (16%) | 630 (15%) |  | 9,157 (16%) | 630 (16%) |  | 551,270 (16%) | 586,408 (16%) |  |
| **Charlson score category** |  |  | 0.40 |  |  | 0.16 |  |  | 0.14 |
| No visits in prior year | 7,671 (13%) | 125 (3.1%) |  | 7,671 (13%) | 125 (7.8%) |  | 419,085 (12%) | 279,049 (7.8%) |  |
| Score 0 | 39,155 (62%) | 2,647 (62%) |  | 39,155 (62%) | 2,647 (64%) |  | 2,182,429 (62%) | 2,297,304 (64%) |  |
| Score 1 | 8,133 (12%) | 749 (17%) |  | 8,133 (12%) | 749 (13%) |  | 446,424 (13%) | 482,110 (13%) |  |
| Score 2 | 3,276 (5.0%) | 370 (8.4%) |  | 3,276 (5.0%) | 370 (5.6%) |  | 182,307 (5.2%) | 199,918 (5.6%) |  |
| Score 3+ | 5,034 (7.5%) | 428 (9.7%) |  | 5,034 (7.5%) | 428 (8.8%) |  | 268,517 (7.7%) | 312,894 (8.8%) |  |
| **Chronic obstructive pulmonary disease** | 6,514 (9.7%) | 582 (13%) | 0.11 | 6,514 (9.7%) | 582 (11%) | 0.04 | 347,546 (9.9%) | 388,816 (11%) | 0.03 |
| **Chronic kidney disease** | 2,960 (4.4%) | 246 (5.5%) | 0.05 | 2,960 (4.4%) | 246 (4.9%) | 0.03 | 155,094 (4.4%) | 175,862 (4.9%) | 0.02 |
| **Atheroscleorotic cardiovascular disease** | 7,195 (11%) | 769 (18%) | 0.19 | 7,195 (11%) | 769 (12%) | 0.02 | 406,301 (12%) | 425,290 (12%) | 0.01 |
| **Cancer/Malignancies** | 1,495 (2.3%) | 196 (4.5%) | 0.12 | 1,495 (2.3%) | 196 (3.3%) | 0.06 | 84,570 (2.4%) | 116,343 (3.3%) | 0.05 |
| **Diabetes** | 6,447 (9.6%) | 441 (9.8%) | 0.00 | 6,447 (9.6%) | 441 (9.6%) | 0.00 | 337,823 (9.7%) | 343,536 (9.6%) | 0.00 |
| **Body mass index category** |  |  | 0.47 |  |  | 0.18 |  |  | 0.15 |
| 18.5 - <25 | 15,581 (26%) | 1,281 (31%) |  | 15,581 (26%) | 1,281 (27%) |  | 910,036 (26%) | 961,658 (27%) |  |
| 25 - <30 | 17,390 (28%) | 1,476 (34%) |  | 17,390 (28%) | 1,476 (30%) |  | 989,570 (28%) | 1,083,245 (30%) |  |
| 30 - <40 | 16,129 (24%) | 1,174 (26%) |  | 16,129 (24%) | 1,174 (27%) |  | 853,304 (24%) | 947,381 (27%) |  |
| >= 40 | 3,858 (5.2%) | 254 (5.4%) |  | 3,858 (5.2%) | 254 (5.3%) |  | 183,351 (5.2%) | 189,048 (5.3%) |  |
| Unknown | 10,311 (17%) | 134 (3.2%) |  | 10,311 (17%) | 134 (11%) |  | 562,502 (16%) | 389,942 (11%) |  |
| **Immunity status at baseline** |  |  | 0.54 |  |  | 0.28 |  |  | 0.25 |
| Neither | 14,601 (22%) | 195 (4.3%) |  | 14,601 (22%) | 195 (13%) |  | 720,428 (21%) | 458,920 (13%) |  |
| Prior infection only | 1,604 (2.4%) | 81 (1.8%) |  | 1,604 (2.4%) | 81 (5.0%) |  | 82,603 (2.4%) | 178,472 (5.0%) |  |
| Vaccinated and prior infection | 3,180 (5.0%) | 326 (7.4%) |  | 3,180 (5.0%) | 326 (7.2%) |  | 182,011 (5.2%) | 257,158 (7.2%) |  |
| Vaccinated only | 43,884 (71%) | 3,717 (86%) |  | 43,884 (71%) | 3,717 (75%) |  | 2,513,721 (72%) | 2,676,725 (75%) |  |
| **Vaccination series at baseline** |  |  | 0.62 |  |  | 0.16 |  |  | 0.12 |
| Not vaccinated | 16,247 (24%) | 289 (6.4%) |  | 16,247 (24%) | 289 (19%) |  | 806,138 (23%) | 667,483 (19%) |  |
| Partial | 2,504 (3.8%) | 43 (0.9%) |  | 2,504 (3.8%) | 43 (2.8%) |  | 125,733 (3.6%) | 100,174 (2.8%) |  |
| Primary series complete | 39,680 (64%) | 3,162 (73%) |  | 39,680 (64%) | 3,162 (69%) |  | 2,259,042 (65%) | 2,454,131 (69%) |  |
| Primary series + additional dose | 4,838 (8.0%) | 825 (19%) |  | 4,838 (8.0%) | 825 (9.8%) |  | 307,851 (8.8%) | 349,486 (9.8%) |  |
| **Vaccine type at baseline** |  |  | 0.54 |  |  | 0.16 |  |  | 0.13 |
| Ad26.COV2.S (Janssen) | 3,000 (4.7%) | 192 (4.4%) |  | 3,000 (4.7%) | 192 (4.2%) |  | 164,981 (4.7%) | 148,658 (4.2%) |  |
| BNT162b2 (Pfizer/BioNTech) | 27,790 (45%) | 2,107 (49%) |  | 27,790 (45%) | 2,107 (45%) |  | 1,578,260 (45%) | 1,610,704 (45%) |  |
| mRNA-1273 (Moderna/NIH) | 16,232 (26%) | 1,731 (40%) |  | 16,232 (26%) | 1,731 (32%) |  | 949,384 (27%) | 1,144,430 (32%) |  |
| Unvaccinated | 16,247 (24%) | 289 (6.4%) |  | 16,247 (24%) | 289 (19%) |  | 806,138 (23%) | 667,483 (19%) |  |

KPNC = Kaiser Permanente Northern California; SMD = standardized mean difference

Sampling weights were calculated as the inverse of the probability of selection in a given age and race strata. We adjusted for non-response bias by calculating a response weight defined as the inverse of the probability of having completed the baseline serology exam and survey. This probability was calculated using the “SuperLearner” package for the R programming language. SuperLearner evaluates several candidate algorithms for model fitting and creates a weighted ensemble model from these candidate algorithms. The algorithms considered in our weighting process were: lasso regression, logistic regression, Pearson’s correlation coefficient, generalized additive models, random forest, gradient boosted decision trees, and a simple mean as a baseline. Each algorithm was fitted with the complete covariate set as well as reduced covariate sets determined by screeners for relationships with the outcome, specifically the univariate Pearson correlation, LASSO regression, and random forest variable importance measures.

The variables used in non-response weighting were race/ethnicity, gender, KPNC service region, age group, Charlson comorbidity score category, binary ever received a COVID-19 vaccine, recruitment wave, body mass index category, baseline immunity status, and multi-level vaccination status. Non-response weights were normalized to the size of the eligible sampled population and trimmed such that the maximum trimmed weight was the 99th percentile of the untrimmed weights. The final weight used in the analysis was the product of the sampling weight and the non-response weight.
